# Will Mobile-Bearing Total Knee Arthroplasty Be Lost to History? A Comparative Study of Long-Term Follow-Up

**DOI:** 10.3390/life14101344

**Published:** 2024-10-21

**Authors:** Sangrim Kim, Joseph Yang, Seokhwan Moon, Sungwook Choi

**Affiliations:** 1 Department of Orthopaedic Surgery, Jeju National University Hospital, School of Medicine, Jeju National University, Jeju 63241, Republic of Korea; 2 Harrow School, 5 High Street, Harrow on the Hill, Middlesex, London HA1 3HP, UK

**Keywords:** total knee arthroplasty, mobile-bearing, fixed-bearing, osteolysis, aseptic loosening

## Abstract

Mobile-bearing (MB) total knee arthroplasty (TKA) implants were introduced as an alternative to fixed-bearing (FB) implants because of their theoretical advantages related to femorotibial rotational mismatch. The purpose of this study is to compare the clinical and radiologic outcomes of MB and FB TKA after approximately 13 years of follow-up. We compared the results of 88 patients with a mean age of 66 years who had received a rotating platform MB implant or a FB implant. The mean follow-up was 13.6 years. The patients were assessed clinically (VAS, ROM, KSS, WOMAC scores) and radiographically before and 13 years after operation. There were no statistically significant differences between the FB and MB groups in terms of clinical outcomes and radiological outcomes (*p* > 0.05 for all). Although the incidence of complications was higher in the MB group, the findings were not statistically significant compared to the FB group (*p* > 0.05 for all). Although there were no significant differences in the clinical and radiologic outcomes between the FB and MB groups, the possible higher risk of osteolysis or aseptic loosening in the MB group could be an important clinical implication when selecting the TKA implant.

## 1. Introduction

Total knee arthroplasty (TKA) is one of the most reliable procedures to relieve pain and restore function in patients with degenerative joint disease [1,2,3,4,5,6,7]. The outcome of TKA is determined by a complex interaction among the bony alignment, the geometry of the implant components, and the soft tissue structures that surround the joint. This interaction, therefore, determines the stability, contact stress, wear, and range of motion [8].

The clinical success rates of fixed-bearing (FB) TKA have been reported to be more than 90% at a minimum 10-year follow-up in patients with end-stage osteoarthritis [6,7,9]. However, conventional FB TKA designs have been associated with problems of polyethylene wear and osteolysis, which can consequently lead to implant loosening and failure. To address these issues, mobile-bearing (MB) designs were introduced as an alternative to fixed-bearing designs [10,11].

In theory, the MB design of TKA can offer more congruent bearing surfaces with a large contact area, decrease contact stress by allowing movement of the insert relative to the tibial tray, and therefore decrease polyethylene wear and osteolysis, subsequently resulting in a lower rate of loosening and presumably an improved range of motion and function than those of a FB TKA [10,12,13,14,15]. 

However, these proposed clinical advantages of the MB TKA have not been consistently supported by the results of randomized controlled trials (RCTs) and meta-analyses, many of which demonstrated inconclusive and contrasting results [15].

The purpose of this study was to compare the clinical and radiological outcomes of fixed-bearing and mobile-bearing TKA. This comparison was accomplished using a series of 88 cases where a mean of 13 years of follow-up was available.

## 2. Materials and Methods

### 2.1. Study Design and Patients

**Figure 1:**
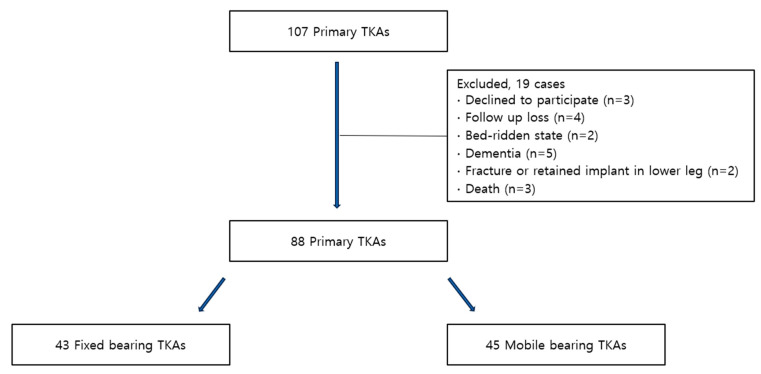
Flow chart showing exclusion and allocation of primary total knee arthroplasty (TKA) cases into fixed-bearing TKAs and mobile-bearing TKAs cohorts.

From July 2007 to December 2010, 107 patients who underwent primary TKA for osteoarthritis (OA) at Jeju National University Hospital (JNUH) using the same perioperative protocol were reviewed for this study. This study was approved by the Institutional Review Board of Jeju National University Hospital (IRB No. 2021-03-15). All eligible patients were provided written informed consent for this study. The inclusion criteria were patients who underwent TKA with a the diagnosis of degenerative osteoarthritis and no history of surgery in the lower leg. The exclusion criteria were having had a previous fracture, operation, or retained implants in the lower leg, or osteomyelitis or a previous intra-articular infection of the knee, metastatic cancer, or a major neurological or musculoskeletal disorder that would affect standing weight bearing or gait. All operations were performed by one of two senior orthopedic surgeons using one of these instrument systems, the NexGen LPS-Flex (Zimmer Inc., Warsaw, IN, USA) or the PFC Sigma RP-F mobile (DePuy Orthopaedics Inc., Warsaw, IN, USA), all of which included posterior cruciate substituting (PS)-type implants fixed with cement. The implant type of each primary TKA included was identified and recorded to classify the cases into target implant categories. We performed a retrospective review with the initial assessment data and prospectively collected the data after approximately a 13-year follow-up. Of the 107 patients who were eligible in this study, a total of 19 patients were excluded, 3 who declined to participate, 4 who were lost to follow-up, 2 who were in a bed-ridden state, 5 who were diagnosed with dementia, 2 who had a fracture or retained an implant in the lower leg, and 3 who died. This resulted in a total of 88 remaining patients for analysis; 43 had undergone TKA with a fixed-bearing insert, and 45 had a mobile-bearing insert (Figure 1).

Even though the patients were mostly women, the patient demographics showed no significant differences between the FB and MB groups. The mean age of all the patients was 66 years (67.42 ± 7.57 year for the FB group, and 65.29 ± 5.65 for the MB group), and the mean body mass index (BMI) was 27.4 kg/m^2^ (26.36 ± 5.45 for the FB group, and 28.01 ± 3.67 for the MB group). The mean follow-up was 13.6 years (13.49 ± 1.91 years for the FB group, and 13.84 ± 1.46 years for the MB group) (Table 1).

### 2.2. Surgical Procedure

The operative techniques and postoperative rehabilitation were the same in both the groups. A pneumatic tourniquet at a pressure of 250 mmHg was applied during the procedure. The surgical procedure was performed using a medial parapatellar approach. The femur underwent intramedullary alignment, and the extramedullary alignment of the tibia was performed. The anterior and posterior cruciate ligaments were resected, and posterior-stabilized (PS)-type implants were used in both the groups. Appropriate medial and lateral balancing procedures were performed in both the groups to achieve equal flexion and extension gaps with a measured resection technique. The patella was selectively resurfaced when there was patellofemoral joint arthritis causing anterior knee pain. Following suction-assisted pulsed lavage, cement drying, and cement pressurization, all the implants were cemented.

### 2.3. Postoperative Care

Continuous passive motion commenced on the second postoperative day for thirty minutes, followed by active range-of-motion exercises, full-weight-bearing standing, and walker-assisted ambulation under the supervision of a physical therapist.

### 2.4. Clinical Outcome Measures

We performed a retrospective review with the collected data from the patients included in this study. All the patient records were examined by two senior residents in the Department of Orthopedic Surgery at Jeju National University Hospital, who were blinded to the implant type used, to establish the functional and radiologic outcomes. All the patients were examined at initial consultation for the operation, and then the last follow-up assessment was performed between April 2021 and December 2023.

Maximum active flexion was recorded with a goniometer, which was defined as maximum extension subtracted from maximum flexion. The Visual Analogue Scale (VAS), the Knee Society Score (KSS), and the Western Ontario and McMaster Universities Osteoarthritis Index (WOMAC) were recorded preoperatively and postoperatively with a standardized questionnaire. The questionnaires were completed by interviewing all the included patients who had undergone TKA with two senior residents in the Department of Orthopedic Surgery.

Preoperative and postoperative radiographic assessment included observing the standing anteroposterior (AP) and lateral views of both knees, a Merchant view of the patella, and a full-length standing hip–knee–ankle view to assess overall limb alignment and component positioning. The pre- and postoperative mechanical axis was defined as the angle between the femoral and tibial mechanical axes from the hip–knee–ankle view. For the last follow-up radiological assessment, the alpha (α), beta (β), gamma (γ), and delta (δ) angles of the components in postoperative AP and lateral radiographs were measured. In addition, the presence of osteolysis, radiolucency, or radiolucent lines at the bone–cement interface was evaluated based on the guidelines of the Knee Society Roentgenographic Evaluation System [16,17,18]. An implant loosening was defined as a change in implant position and/or circumferential radiolucent lines thicker than 2 mm in all zones. All radiologic parameters were analyzed and recorded twice at two-week intervals by two senior residents who were blinded to the study. The intraclass and interclass correlations for each parameter were acceptable (>0.8).

### 2.5. Statistical Methods

All statistical analyses were performed using web-based analysis with the R 4.3.1 free version (https://web-r.org). Independent *t*-tests and paired *t*-tests were performed for continuous variables. A chi-squared test with continuity correction was performed for categorical variables. A *p*-value < 0.05 was considered statistically significant.

## 3. Results

### 3.1. Clinical Results

**Figure 2:**
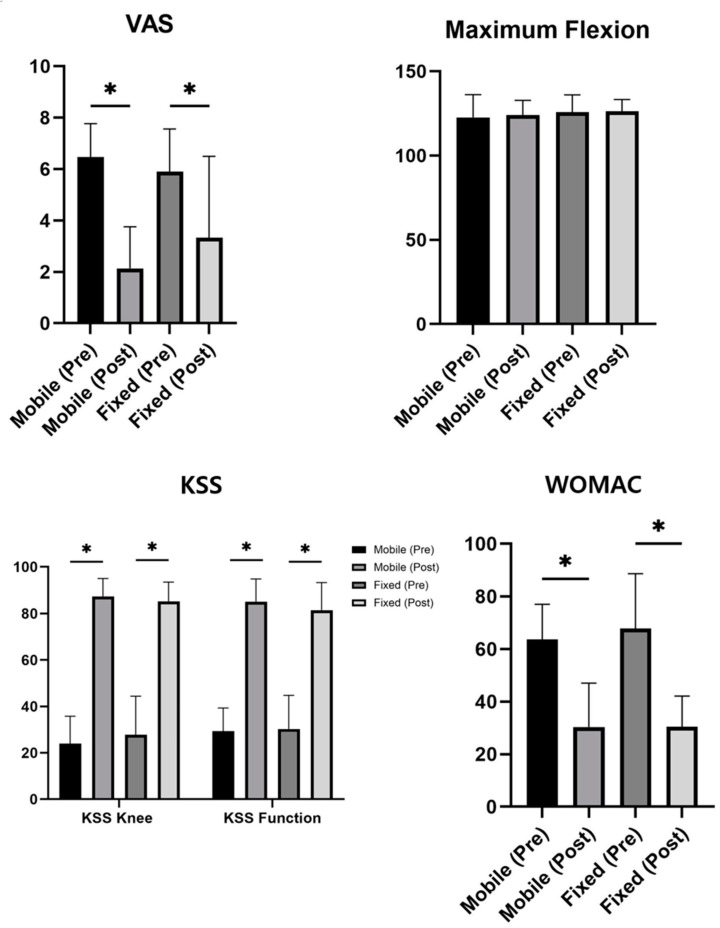
Summary of patient-reported outcome measures at preoperative and last follow-up. *: *p* < 0.05. VAS: Visual Analogue Scale; KSS: Knee Society Score; WOMAC: Western Ontario and McMaster Universities Osteoarthritis Index.

A comparison of the clinical outcomes between the two groups at the preoperative and last follow-up is shown in Table 2 and Figure 2.

All the patients were reviewed at a mean of 13 years of follow-up (13.49 ± 1.91 for the FB group and 13.84 ± 1.46 for the MB group, *p* = 0.327).

The mean VAS score decreased from 5.88 ± 1.67 points in the FB group and 6.44 ± 1.33 points in the MB group preoperatively (*p* = 0.090) to 3.33 ± 3.17 points in the FB group and 2.53 ± 3.12 points in the MB group at the time of the last follow-up (*p* = 0.240), respectively.

There were no significant differences between the two groups in terms of preoperative knee flexion contracture and maximum knee flexion: 7.67 ± 5.91° and 125.81 ± 10.23° for the FB group, and 8.78 ± 6.84° and 122.67 ± 13.51° for the MB group, respectively (*p* = 0.421, *p* = 0.223). At the last follow-up, knee flexion contracture improved to 3.37 ± 5.64° for the FB group and 3.56 ± 4.96° for the MB group, while maximum knee flexion was similar to the preoperative value (*p* > 0.05, paired *t*-test).

Both the mean scores of KSS (clinical) and KSS (functional) increased from 27.81 ± 16.60 and 30.23 ± 14.51 points in the FB group and 23.96 ± 11.82 and 29.33 ± 9.98 points in the MB group preoperatively (*p* = 0.215; *p* = 0.737) to 85.28 ± 8.25 and 81.47 ± 11.85 points in the FB group and 87.38 ± 7.71 and 85.13 ± 9.73 points in the MB group at the time of the last follow-up (*p* = 0.221; *p* = 0.115), respectively.

The preoperative WOMAC total scores (67.81 ± 20.84 points in the FB group and 63.67 ± 13.38 points in the FB group; *p* = 0.273) were improved significantly (30.44 ± 11.70 points in the FB group and 30.33 ± 16.72 in the MB group; *p* = 0.972) in both the groups at the time of the last follow-up. Even though the functional mean scores of KSS and WOMAC at the latest follow-up revealed the results of 85.13 ± 9.73 and 23.56 ± 10.06 in the MB group, being slightly better than the results of 81.47 ± 11.85 and 26.49 ± 10.59 in the FB group, it was not statistically significant (*p* = 0.115; *p* = 0.186).

Overall, the outcome parameters, including the VAS, the KSS, and the WOMAC score, improved significantly after FB TKA or MB TKA (*p* < 0.05 for all); however, they were not significantly different between the two groups either preoperatively, or at the time of the last follow-up (*p* > 0.05 for all).

### 3.2. Radiographic Results

A comparison of the radiographic outcomes between the groups at the preoperative and last follow-up is shown in Table 3.

The results of the mean mechanical axis, alpha, beta, gamma, and delta angles of the FB and MB groups are given in Table 3. There were no statistically significant differences between the two groups in terms of the mechanical axis preoperatively (11.49 ± 6.09° in the FB group and 12.00 ± 4.17° in the MB group; *p* = 0.651) and at the last follow-up (2.40 ± 3.86° in the FB group and 1.61 ± 2.59° in the MB group; *p* = 0.267). Similarly, there were no significant differences between the two groups with respect to the alignment of the femoral and tibial components in the coronal and sagittal planes. From the anteroposterior view, the mean femoral component angle was 95.62 ± 2.59° in the FB group and 95.87 ± 1.76° in the MB group (*p* = 0.6), and the mean tibial component angle was 90.81 ± 3.59° in the FB group and 90.40 ± 2.43° in the MB group (*p* = 0.532). On the lateral radiograph, the mean femoral component angle was 4.26 ± 0.57° in the FB group and 4.08 ± 0.76° in the MB group (*p* = 0.193), and the mean tibial component angle was 84.24 ± 2.77° in the FB group and 83.50 ± 2.11° in the MB group (*p* = 0.161).

### 3.3. Complications and Reoperation

The complications and reoperation rates are shown in Table 4.

The aseptic loosening of the tibial component was found in two patients (4.44%) in the MB group, and both the cases subsequently underwent revision TKA. Both the cases of aseptic loosening had severe preoperative varus deformity over 14° and 21° each, and revision was performed 6 years and 4 months and 13 years and 2 months after surgery, respectively.

Meanwhile, even though two knees (4.44%) in the FB group had tibial radiolucent lines (<1 mm in width) in zone 1, this did not result in a second procedure. As for the polyethylene (PE) wear, there was one case in the FB and MB groups each (2.33% and 2.22%), and both of them required the PE to be changed.

Overall, even though the incidence of complications and reoperations for any reason was higher in the MB group, the findings were not statistically significant compared to those in the FB group (*p* > 0.05 for all).

## 4. Discussion

The objective of this retrospective study comparing mobile and fixed-bearing TKA was to ascertain if the type of bearing significantly influenced the clinical and radiological outcomes. The most important finding of the present study was the absence of a difference between mobile and fixed-bearing TKA in terms of the clinical and radiological outcomes.

In theory, the mobile-bearing (MB) design of TKA can offer more congruent bearing surfaces with a large contact area, decrease contact stress by allowing for the movement of the insert relative to the tibial tray, and therefore decrease polyethylene wear and osteolysis, subsequently resulting in a lower rate of loosening and a presumably more improved range of motion and function than those of fixed-bearing (FB) TKA [10,12,13,14,15].

However, we were not able to find a difference between the patients with these implants with regards to the level of pain, the range of motion, and the functional outcome as measured by the KSS and WOAMC at a mean of 13 years of follow-up. Even though both the groups had good-to-excellent pain relief and WOMAC scores and KSSs, the clinical evaluation of these scores did not reveal any statistically significant differences between the fixed-bearing and mobile-bearing groups at the last follow-up (VAS: *p* = 0.240; KSS, clinical: *p* > 0.221; KSS, function: 0.115; WOMAC total: *p* > 0.972).

The greater range of motion resulting from mobile bearings was not apparent in this study, and the result is consistent with the conclusions of previous studies [9,14,17,18,19,20,21,22]. To be specific, there was no difference between the patients with mobile-bearing TKA and those with fixed-bearing TKA in terms of maximum knee flexion at a mean of 13 years postoperatively (*p* = 0.064). Most et al. reported similar kinematic patterns of posterior femoral translation and tibiofemoral rotation between the FB and MB TKA groups, suggesting that the mobile insert stops moving at just under 90 degrees of flexion and performs as a fixed-bearing insert afterwards [9]. Even though the results of maximum knee flexion in this study were not reviewed routinely, they are probably in agreement with the kinematic findings, as there was no difference between the results at the initial consultation preoperatively and at a mean of 13 years of follow-up postoperatively (*p* > 0.05).

In addition, even though the functional mean scores of the KSS and the WOMAC at the last follow-up revealed results of 85.13 ± 9.73 and 23.56 ± 10.06 in the MB group, being slightly better than results of 81.47 ± 11.85 and 26.49 ± 10.59 in the FB group, they were not statistically significant (*p* = 0.115; *p* = 0.186). These findings are supported by previous studies that also do not demonstrate any significant differences between FB and MB TKA in the functional outcomes [19,23,24,25,26,27].

**Figure 3:**
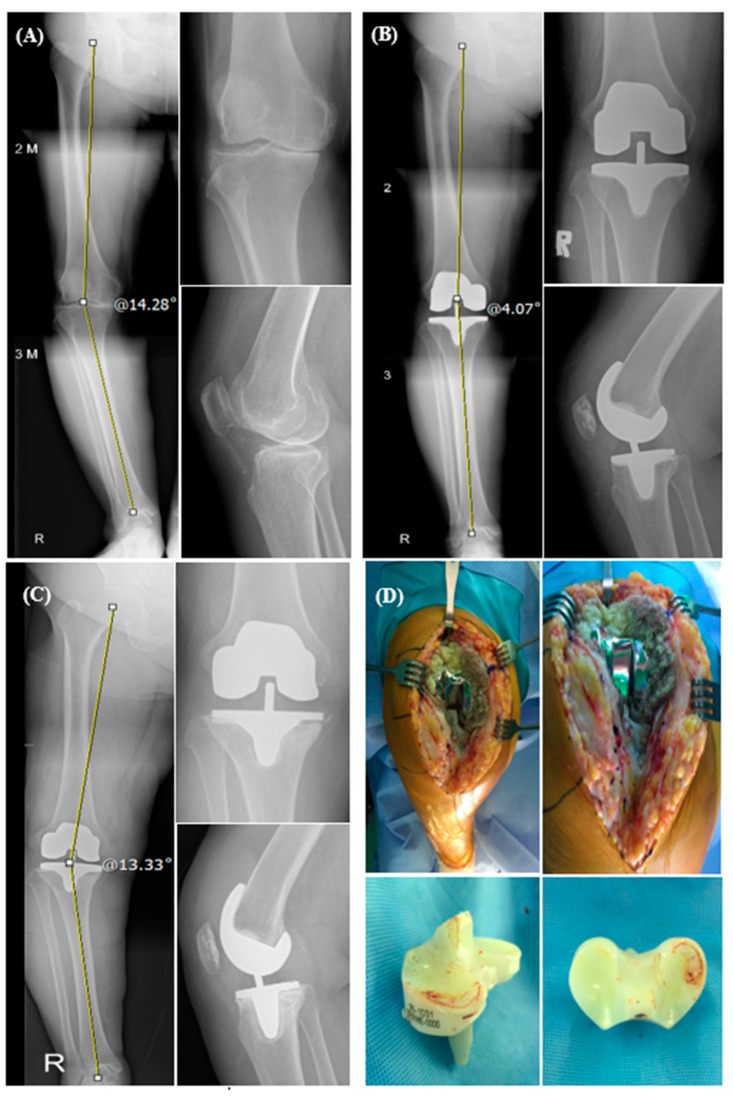
X-rays and photos of a 64-year-old woman. (**A**) Preoperative varus was 14.28°. (**B**) Postoperative alignment was acceptable at 4.07°. (**C**) Six years and two months after surgery, tibial component loosening was found. (**D**) Revision was performed 6 years and 4 months after surgery.

Meanwhile, the mobile-bearing design theoretically could reduce bone-implant stress at the tibial surface [25,26], and therefore reduce the risk of loosening [28,29]. Nevertheless, there were 4.44% more cases of aseptic tibial component loosening in the MB group, and both the cases underwent subsequent revision TKA. One of the important causes of revision total knee arthroplasty (TKA) is tibial component loosening [30,31], especially on the tibial side [32,33]. According to earlier research, patients with more-severe varus deformity (>8° of varus) had a much greater risk of tibial component loosening than the patients with less varus deformity. This is probably due to the technical difficulty in soft tissue balancing and the high strain on the tibial component in the severe varus knee [34]. Two cases of aseptic tibial component loosening in the MB group in this study also had a severe varus deformity (21.03° and 14.28°, respectively) preoperatively, and consequently underwent a second procedure at the times of 13 years of follow-up and 6 years of follow-up, respectively (one of the cases is shown in Figure 3). Although the anatomic alignment for this primary TKA case was corrected to 4° of varus, it would be a challenge to determine whether the alignment should be corrected more with the mechanical axis for the patient with severe varus deformity in order to prevent progressive alignment changes or deformity after TKA. The severe varus knee in this study might have been the key factor resulting in aseptic loosening and consequent revision TKA. However, although there was a higher incidence of osteolysis or tibial loosening in the MB group compared to that in the FB group, it was not statistically significant (*p* = 0.495). Nevertheless, the potential higher risk of osteolysis or aseptic loosening in the MB group would be an important clinical implication when selecting the TKA implant, particularly for the patient with severe varus deformity.

As for the complication at a minimum of 13 years follow-up postoperatively, even though the incidence of complications and reoperations for any reason was higher in the MB group, the findings were not statistically significant compared to those in the FB group (*p* > 0.05 for all). These findings are supported by the previous studies that also do not demonstrate any significant differences between FB and MB TKA in the complication and reoperation rates [26,27].

Taking the factors mentioned above into consideration, the mean of the 13 years follow-up findings in this study do not support the clinical advantage of mobile-bearing (MB) design TKA as there were no statistically significant differences between the FB and the MB TKA groups in terms of the clinical outcomes, including the VAS, the KSS, the WOMAC scores, and the radiological outcomes (*p* > 0.05 for all).

This study had several limitations. First, this retrospective study design could introduce selection bias due to the lack of randomization. Second, although the two groups were similar in age, sex, underlying diseases, and clinical baseline characteristics, all the patients who underwent total knee arthroplasty were of Asian ethnicity, and the majority of whom were female. Third, the present study used fixed- and mobile-bearing implants manufactured by different companies, which may have influenced our results as the two prostheses from different companies have different features. Forth, this study is a single-center investigation with a relatively small number of cases of TKA, and thus is limited in generalizability and may have been underpowered to detect the clinical and radiological difference between the two groups. Fifth, the clinical outcomes measured with the VAS, the KSS, and the WOAMC may not be sensitive enough to find subtle differences in the level of pain and function because most of the patients included in this study were local people who spoke in an uncommon dialect. Therefore, it might have been difficult to detect and elucidate the patient’s expression and meanings about the pain or functional level.

Therefore, the conclusions of this study should be considered with these limitations. Although the results of the present study indicated that the type of bearing in TKA does not significantly influence the clinical and radiological outcomes, the possible higher risk of osteolysis or aseptic loosening should be carefully considered for the patient with severe varus deformity when selecting the MB implant.

## 5. Conclusions

In this clinical retrospective study, there were no significant differences in the clinical and radiologic outcomes between the fixed-bearing and mobile-bearing TKA implants at a mean of a 13-year follow-up. However, the possible higher risk of osteolysis or aseptic loosening in the MB group would be an important clinical implication when selecting the TKA implant, especially for those who have severe varus deformity.

## Figures and Tables

**Table 1:** The demographics and baseline characteristics of the fixed-bearing and the mobile-bearing groups *.

	Fixed *	Mobile *	*p*
	(N = 43)	(N = 45)	
Sex			0.385
Male	1 (2.33%)	4 (8.89%)	
Female	42 (97.67%)	41 (91.11%)	
Age (years)	67.42 ± 7.57	65.29 ± 5.65	0.176
Height (m)	1.53 ± 0.06	1.54 ± 0.06	0.664
Weight (kg)	61.86 ± 12.71	66.28 ± 9.14	0.066
BMI (kg/m^2^)	26.36 ± 5.45	28.01 ± 3.67	0.102
Operation side			1.000
Right	21 (48.84%)	21 (46.67%)	
Left	22 (51.16%)	24 (53.33%)	
Hypertension	25 (58.14%)	29 (64.44%)	0.698
Hyperlipidemia	3 (6.98%)	3 (6.67%)	1.000
Osteoporosis	4 (9.30%)	7 (15.56%)	0.573
Kidney disease	2 (4.65%)	4 (8.89%)	0.715
Liver cirrhosis	0	0	
Depression	1 (2.33%)	2 (4.44%)	1.000

* The values are given as the mean and the standard deviation, or the number of patients (%).

**Table 2:** Comparison of clinical outcomes between groups at preoperative and last follow-up.

	Fixed *	Mobile *	*p*
	(N = 43)	(N = 45)	
VAS			
Preoperative	5.88 ± 1.67	6.44 ± 1.33	0.090
At latest follow-up	3.33 ± 3.17	2.53 ± 3.12	0.240
Flexion contracture			
Preoperative	7.67 ± 5.91	8.78 ± 6.84	0.421
At latest follow-up	3.37 ± 5.64	3.56 ± 4.96	0.871
Maximum knee flexion			
Preoperative	125.81 ± 10.23	122.67 ± 13.51	0.223
At latest follow-up	126.28 ± 7.08	124.00 ± 8.83	0.186
KSS (clinical)			
Preoperative	27.81 ± 16.60	23.96 ± 11.82	0.215
At latest follow-up	85.28 ± 8.25	87.38 ± 7.71	0.221
KSS (function)			
Preoperative	30.23 ± 14.51	29.33 ± 9.98	0.737
At latest follow-up	81.47 ± 11.85	85.13 ± 9.73	0.115
WOMAC (pain)			
Preoperative	10.79 ± 4.18	9.42 ± 3.33	0.092
At latest follow-up	2.09 ± 2.83	4.82 ± 13.29	0.185
WOMAC (stiffness)			
Preoperative	5.02 ± 2.32	5.38 ± 1.25	0.379
At latest follow-up	1.86 ± 1.15	1.96 ± 1.07	0.688
WOMAC (function)			
Preoperative	52.00 ± 15.53	49.13 ± 9.35	0.300
At latest follow-up	26.49 ± 10.59	23.56 ± 10.06	0.186
WOMAC (total)			
Preoperative	67.81 ± 20.84	63.67 ± 13.38	0.273
At latest follow-up	30.44 ± 11.70	30.33 ± 16.72	0.972

* The values are given as the mean and the standard deviation. Preoperative: preoperative baseline data; VAS: Visual Analogue Scale; KSS: Knee Society Score; WOMAC: Western Ontario and McMaster Universities Osteoarthritis Index.

**Table 3:** Comparison of radiographic outcomes between groups at preoperative and last follow-up.

	Fixed *	Mobile *	*p*
	(N = 43)	(N = 45)	
HKA angle (°)			
Preoperative	11.49 ± 6.09	12.00 ± 4.17	0.651
At latest follow-up	2.40 ± 3.86	1.61 ± 2.59	0.267
Component position			
α angle (°)	95.62 ± 2.59	95.87 ± 1.76	0.600
β angle (°)	90.81 ± 3.59	90.40 ± 2.43	0.532
γ angle (°)	4.26 ± 0.57	4.08 ± 0.76	0.193
δ angle (°)	84.24 ± 2.77	83.50 ± 2.11	0.161

* The values are given as the mean and the standard deviation. HKA: hip–knee–ankle angle; The alpha (α), beta (β), gamma (γ), and delta (δ) angles of the components in the postoperative AP and lateral radiographs.

**Table 4:** Complications and reoperations.

	Fixed *	Mobile *	*p*
	(N = 43)	(N = 45)	
Aseptic loosening	0 (0.0%)	2 (4.44%)	0.495
Osteolysis	0 (0.0%)	2 (4.44%)	0.495
Polyethylene wear	1 (2.33%)	1 (2.22%)	1.000
Reoperation			
Insert change	1 (2.33%)	1 (2.22%)	1.000
Revision TKA	0 (0.0%)	2 (4.44%)	0.495

* The values given are the number of patients (%).

## Data Availability

The data presented in this study are available on request from the corresponding author.

## Abbreviations

BMI: body mass index; FB: fixed bearing; HKA: hip–knee–ankle angle; KSS: Knee Society Score; MB: mobile bearing; PS: posterior stabilized; TKA: total knee arthroplasty; VAS: Visual Analogue Scale; WOMAC: Western Ontario and McMaster Universities Osteoarthritis Index.
